# Impacts of climate change on the potential distribution of *Pulex simulans* and *Polygenis gwyni*

**DOI:** 10.1002/ece3.11621

**Published:** 2024-07-17

**Authors:** Zihao Wang, Nan Chang, Hongyun Li, Xiaohui Wei, Yuan Shi, Ke Li, Jinyu Li, Chenran Guo, Qiyong Liu

**Affiliations:** ^1^ School of Public Health Nanjing Medical University Nanjing Jiangsu China; ^2^ National Key Laboratory of Intelligent Tracking and Forecasting for Infectious Diseases, National Institute for Communicable Disease Control and Prevention Chinese Center for Disease Control and Prevention Beijing China; ^3^ Department of Infectious Diseases Heze Center for Disease Control and Prevention Heze Shandong China; ^4^ School of Public Health, Cheeloo College Medicine Shandong University Jinan China

**Keywords:** climate change, fleas, MaxEnt, plague vectors, *Polygenis*, potential distribution, *Pulex*

## Abstract

*Pulex simulans* and *Polygenis gwyni* are vectors of many flea‐borne diseases. They were widely recorded in the United States and Mexico between 1970 and 2000. Maximum entropy models were used to explore the habitats of both fleas under different climate scenarios to provide the scientific basis for the surveillance and control of flea‐borne diseases. We screened climate variables by principal component analysis and Pearson's correlation test and evaluated model performance by ROC curve. ArcMap was used to visualize expressions. Under current climatic conditions, the medium and highly suitable areas for *P. simulans* are estimated to be 9.16 × 10^6^ km^2^ and 4.97 × 10^6^ km^2^, respectively. These regions are predominantly located in South America, along the Mediterranean coast of Europe, the southern part of the African continent, the Middle East, North China, and Australia. For *P. gwyni*, the medium and highly suitable areas under current climatic conditions are approximately 4.01 × 10^6^ and 2.04 × 10^6^ km^2^, respectively, with the primary distribution in North China extending to the Himalayas, near the Equator in Africa, and in a few areas of Europe. Under future climate scenarios, in the SSP3‐7.0 scenario for the years 2081–2100, the area of high suitability for *P. simulans* is projected to reach its maximum. Similarly, in the SSP2‐4.5 scenario for 2061–2080, the area of high suitability for *P. gwyni* is expected to reach its maximum. Under global climate change, there is a large range in the potential distribution for both fleas, with an overall upward trend in the area of habitat under future climate scenarios. Governments should develop scientific prevention and control measures to prevent the invasive alien species flea.

## Graphical abstract

*Pulex simulans* and *Polygenis gwyni* are vectors of many flea‐borne diseases. We used the MaxEnt to predict the potential habitats of *P. simulans* and *P. gwyni*.

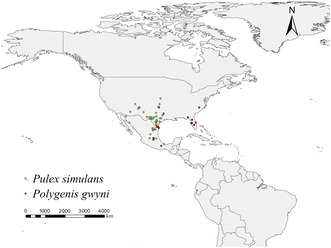

## 1 INTRODUCTION

The advancement of human economic activities has been a significant driver of climate change (Trenberth, 2018), which in turn has heightened the risk of infectious disease transmission. The escalation in global temperatures has broadened the geographical distribution of arthropod vectors, enabling their migration to higher latitudes and altitudes, thereby intensifying the dissemination of vector‐borne diseases (Bale et al., 2002; Githeko et al., 2000). Among these vectors, *P. simulans* and *P. gwyni* are noteworthy for their role in the transmission of plague. *P. simulans* is thought to be able to carry the Yersinia pestis, and *P. gwyni*, a flea species associated with cotton rats, has facilitated the spread of plague to *Sigmodon hispidus* and *Rattus norveicus* in the southern and southwestern United States (Holdenried, 1952; Poje et al., 2020).

In terms of distribution, research by AG Mutlow has highlighted that *P. simulans* exhibits a broad geographic presence across the United States, Central, and South America, with a diverse host range (Mutlow et al., 2006). Similarly, Durden, LA et al.'s study identified the primary habitat of *P. gwyni* in the Carolinas along the southeastern Atlantic coast of the United States (Durden et al., 1999), underscoring the ecological and epidemiological significance of these flea species in the context of disease transmission. Climate factors are closely related to the occurrence of vector‐borne infectious diseases. Climate change greatly affects the behavior of flea species and their ability to transmit diseases (Gage et al., 2008). For example, factors such as temperature, precipitation, and relative humidity can affect the growth and survival of fleas (Guernier et al., 2014), and research by Xu and his team on the plague in China during the 19th and 20th centuries found a nonlinear relationship between the intensity of plague and precipitation (Xu et al., 2011). Climate factors also affect the population numbers and distribution of vectors (Liu et al., 2018), creating new epidemic risks.

Therefore, using ecological niche models, which are particularly suitable for ecology and biogeography, to assess the environmental conditions within a species' distribution range helps predict the possibility of biological invasions and plays an important role in preventing outbreaks of flea‐borne diseases. The Maximum Entropy (MaxEnt) model (Feng et al., 2019; Peterson, 2003) stands out in this domain. MaxEnt, known for its continuous prediction capabilities (Phillips et al., 2006), is adept at generating various predictive metrics such as omission rates, receiver operating characteristic (ROC) curves, and response curves. This model is acclaimed for its exceptional predictive accuracy, as highlighted by Elith et al. (2006), making it a preferred choice in geographic modeling. Further emphasizing MaxEnt's capabilities, one research (Zhang & Wang, 2022) demonstrates its superiority over other models like logistic regression, artificial neural networks, and support vector machines. The MaxEnt model's strengths lie in its ability to account for interactions and the significance of spatial variables, coupled with generating comprehensible response curves, thereby offering a more coherent explanation of ecological phenomena. This robust framework renders MaxEnt an invaluable tool for understanding and predicting the dynamics of species distribution, particularly in the context of changing climatic conditions.

As economic globalization, urbanization, and increases in personal income fuel trade between nations and travel among individuals (Wu et al., 2017), along with climate change, there has been a facilitated widespread dissemination of pathogens across the globe. Consequently, there is an elevated risk of diseases transmitted by fleas. The innovation of this study lies in its exploration of the changes in suitable habitats for the plague bacterium‐carrying vector species *P. simulans* and *P. gwyni*, driven by current and future climate scenarios, through the use of the Maxent model. Although these two fleas have not been found in other areas for the time being, they are at risk of invasion under certain conditions. So, this investigation is crucial for preventing the invasion of these vectors and, by extension, is vital for the prevention of flea‐borne diseases, holding significant public health implications.

## 2 MATERIALS AND METHODS

### 2.1 Distribution point data acquisition

**FIGURE 1:**
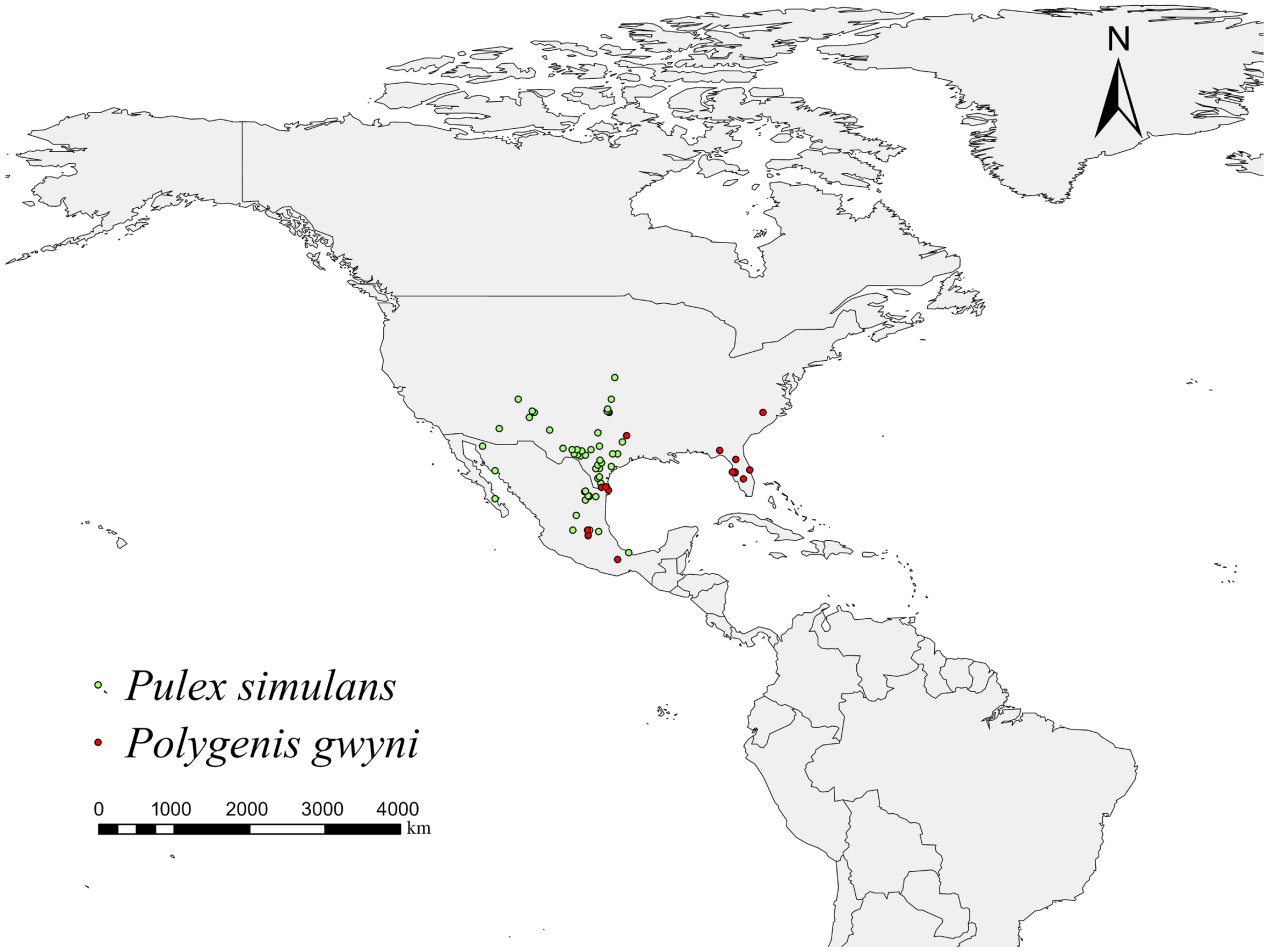
Occurrence points of *Pulex simulans* and *Polygenis gwyni*.

The data of fleas were downloaded from Global Biodiversity Information Facility (https://www.gbif.org; accessed on 23 March 2023). We checked the accuracy of the data by selecting only distribution point data from 1970 to 2000 and deleting blank and duplicate data. In ENMTools software (https://www.activestate.com/products/perl/downloads; accessed on 15 April 2023), click Trim duplicate occurrences, import the csv file with distribution points, and select Grid cell, then we can get the csv file with deleted redundant data in the same grid. Finally, 50 points for *P. simulans* and 17 points for *P. gwyni* were obtained and then stored as a CSV file containing species names, longitude, and latitude. The distribution can be seen in Figure 1.

### 2.2 Environmental data acquisition and analysis

Environmental data from 1970 to 2000 were downloaded from WorldClim (version 2.1, https://worldclim.org/; accessed on: 24 March 2023), including 19 Bioclimatic variables, 36 historical monthly climate data and elevation data, with a spatial resolution of 5 mm. and download environmental data for 2021–2040, 2041–2060, 2061–2080, 2081–2100 for the four Shared Socio‐economic Pathways (SSPs): 126, 245, 370 and 585 scenarios. All environmental data were downloaded in TIFF format and then converted to ASCII format using ArcGIS (version 10.5), purchased by the Chinese Center for Disease Control and Prevention.

The filtered distributions of *P. simulans* and *P. gwyni* and the 56 environmental variables were imported in MaxEnt software (version 3.4.1, https://biodiversityinformatics.amnh.org; accessed on 13 April 2023) and ran once to obtain the variable contributions. These environmental variables with a contribution rate greater than or equal to 2% were screened out. Statistical analyses revealed high correlations among certain environmental variables at distribution points. To clarify the similarities among bioclimatic variables, we initially conducted a Kaiser‐Meyer‐Olkin (KMO) test. A Kaiser‐Meyer‐Olkin Measure of Sampling Adequacy greater than 0.6 indicates the suitability of employing the principal component analysis (PCA). The environmental variables of the distribution points for *P. simulans* yielded a Kaiser‐Meyer‐Olkin Measure of Sampling Adequacy of 0.669, whereas those for *P. gwyni* recorded a value of 0.458. And Bartlett's test of sphericity yields a significance level of <.001. Consequently, we utilized PCA to process the environmental variables at the *P. simulans* distribution points (Table 1) and selected the environmental variables for *P. gwyni* through a correlation significance test (Table 2). Variables with an absolute correlation coefficient >.8 were excluded if they contributed less. Furthermore, to ascertain the presence of multicollinearity, the variance inflation factor (VIF) was calculated for all variables, with all VIF values being below 10.

**TABLE 1:** Results of principal component analysis.

Climate variables	PCA1	PCA2	PCA3
bio2	−0.608	−0.538	0.408
bio3	0.483	−0.782	0.278
bio14	0.264	0.670	−0.521
prec01	0.559	0.563	−0.349
prec07	0.739	−0.220	−0.514
prec08	0.827	−0.192	−0.328
prec09	0.874	0.089	−0.263
tmax02	0.732	−0.203	0.632
tmax07	−0.247	0.685	0.599
tmax08	−0.118	0.747	0.609
tmin01	0.902	−0.013	0.409
tmin02	0.898	0.054	0.416
tmin03	0.882	0.194	0.388

**TABLE 2:** Percentage contributions of climate variables for *Polygenis gwyni.*

Climate variables	Percent contribution
prec9	46
tmin1	25
bio8	13.7
prec4	10
bio4	5.2

### 2.3 Parameter optimization for maximum entropy models

MaxEnt uses a maximum entropy model to predict the likely range of species from known species distribution information and environmental data. However, the predictions of unoptimized models can be subject to significant fit bias, leading to a mis‐assessment of species' ecological niches (Kong et al., 2019). Therefore, the parameters need to be changed to optimize the model. The regularization multiplier has eight levels: 0.5, 1, 1.5, 2, 2.5, 3, 3.5, 4. Feature classes have five parameters including Linear (L), Quadratic (Q), Product (P), Threshold (T), Hinge (H).

R software and DIVA‐GIS correlated the environmental data and adjusted the parameters to optimize the MaxEnt model. Environmental data were correlated using ArcGIS, DIVA‐GIS, and R software. Initially, raster files were exported from ArcGIS in the BIL format. In DIVA‐GIS, by selecting “Data” from the menu and choosing “Import to Gridfile” with the type set to BIL, the BIL format files were converted into grd and gil formats. The best model was determined using the Akaike Information Criterion (AIC) in the ENMeval package in R software, where an optimal model is indicated by a deltaAICc of 0. In this study, the optimal model for *P. simulans* was identified with a regularization multiplier (rm) of 1 and feature class (fc) as QHP; for *P. gwyni*, the optimal model had a rm of 2 and fc as LQH. The training and testing datasets are randomly generated, with 75% of the data points used for training and 25% used for testing. Cross‐validation with the testing and validation sets is utilized to avoid overfitting.

### 2.4 Distribution and change of suitable area

**FIGURE 2:**
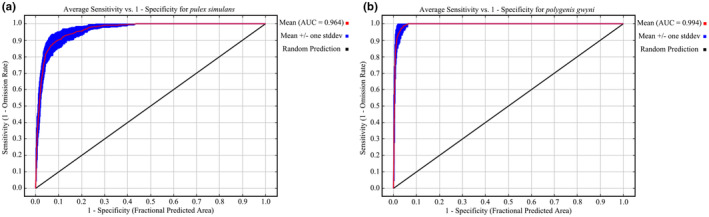
Receiver operating characteristic curve by the MaxEnt for *Pulex simulans* (a) and *Polygenis gwyni* (b).

The suitable area, which refers to the range most conducive to the growth and development of an organism, for *P. simulans* and *P. gwyni* was predicted using MaxEnt software. These results were analyzed using ArcGIS software not only to derive a global map of habitat distribution but also to calculate the area of the habitat. By implementing the reclassify function in ArcGIS, asc files derived from MaxEnt were selected. A natural break point classification (Jenks) was employed, dividing the habitats into non‐suitable, low suitable, medium suitable, and high suitable area. Importantly, the breakpoints under current and future climate conditions were maintained consistently. World map (1:10 million) was available on the Natural Earth website (https://www.naturalearthdata.com/downloads; accessed on 23 March 2023). The ROC curve (Figure 2) was used to test the accuracy of results, and the AUC value >0.9 indicates high accuracy.

## 3 RESULTS

### 3.1 Distribution concerning environmental variables

**FIGURE 3:**
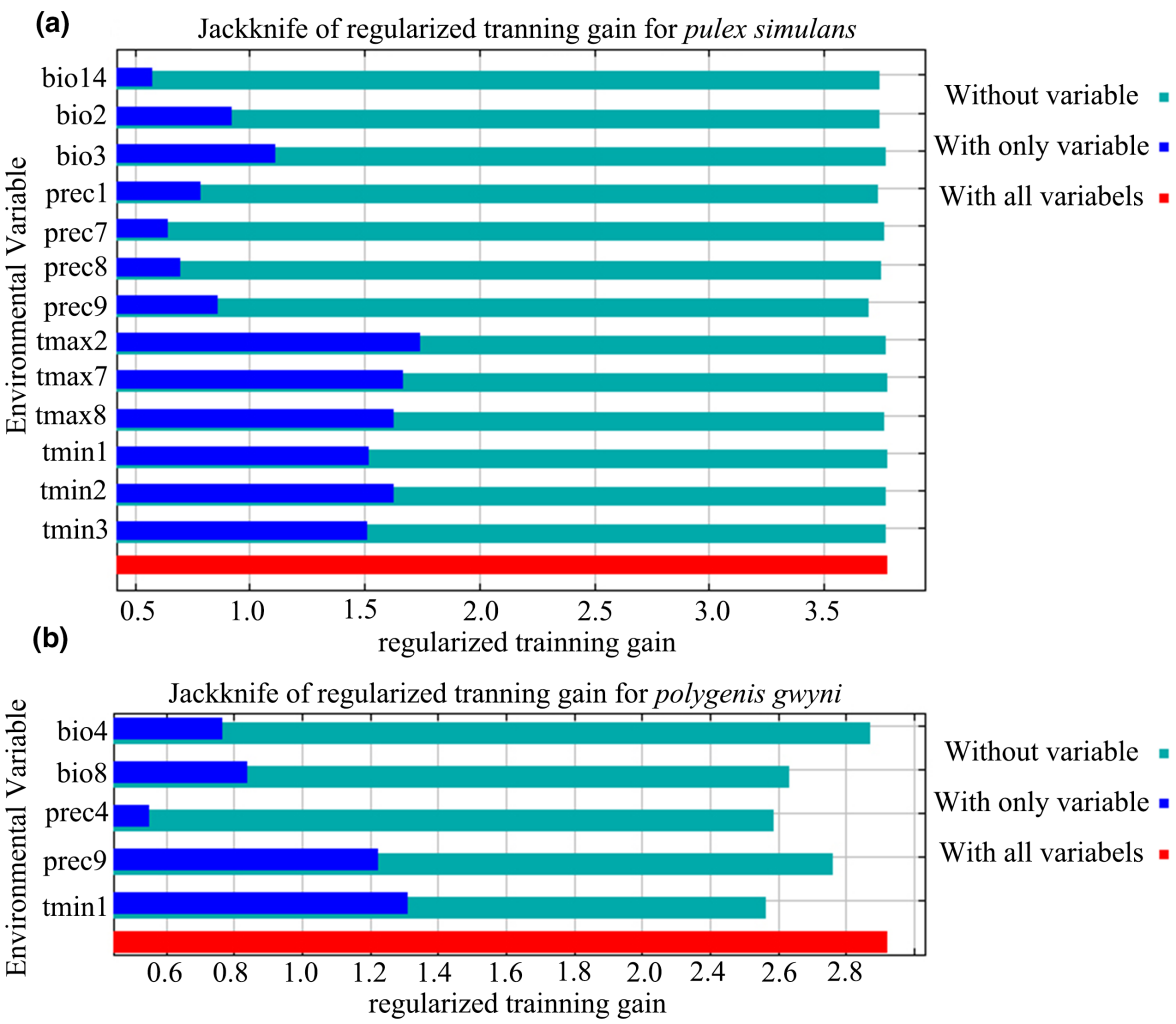
The results of the Jackknife test of variable importance for *Pulex simulans* (a) and *Polygenis gwyni* (b): The green bands indicate the degree of specificity of the variable, with shorter bars indicating that the variable contains unique information and is more likely to influence the distribution of the species. The blue bands indicate the variable's effectiveness on the species' distribution, with longer bars indicating that the variable contains more effective information. All bioclimatic variables utilized in the model are deemed essential.

The ROC curve (Figure 2) is used to test the accuracy of results, and the AUC value greater than 0.9 indicates high accuracy and the Jackknife test (Figure 3) is used to examine the contribution of each variable to influencing distribution (Çoban et al., 2020). We predicted the distribution of potential suitable area for *P. simulans* and *P. gwyni* under current and future climatic conditions by parameter‐optimized maxent. In our study, we identified 13 environmental variables contributing more than 2% to the distribution of *P. simulans* and performed a PCA to synthesize three new variables. Meanwhile, we selected five environmental variables that contributed more than 2% to the distribution of *P. gwyni*, each with a correlation coefficient lower than 0.8. Their VIF values were below 10. These environmental variables were incorporated into a parameter‐optimized MaxEnt model to predict their potential suitable habitat distributions under current and future climate scenarios. In the modeling, it was observed that the key determinants of *P. simulans* habitat are primarily PCA1 (represented mainly by precipitation in August and September) and PCA3 (represented mainly by the highest temperatures in February and July), whereas the crucial factors for *P. gwyni* habitat were identified as September precipitation and the minimum temperature in January.

### 3.2 Distribution of potentially suitable areas under current climate scenarios

**FIGURE 4:**
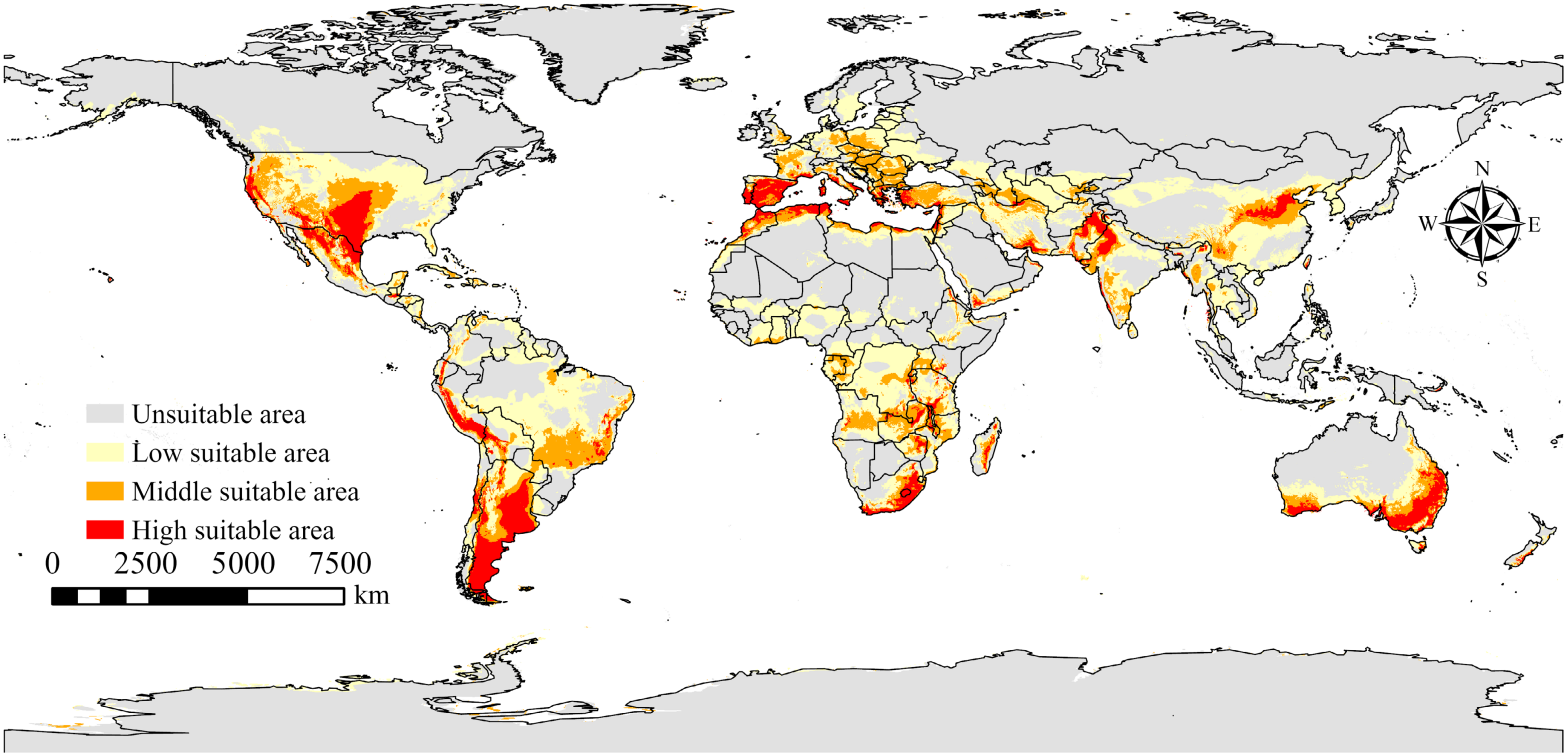
Suitable areas for *Pulex simulans* under current climate scenarios.

**FIGURE 5:**
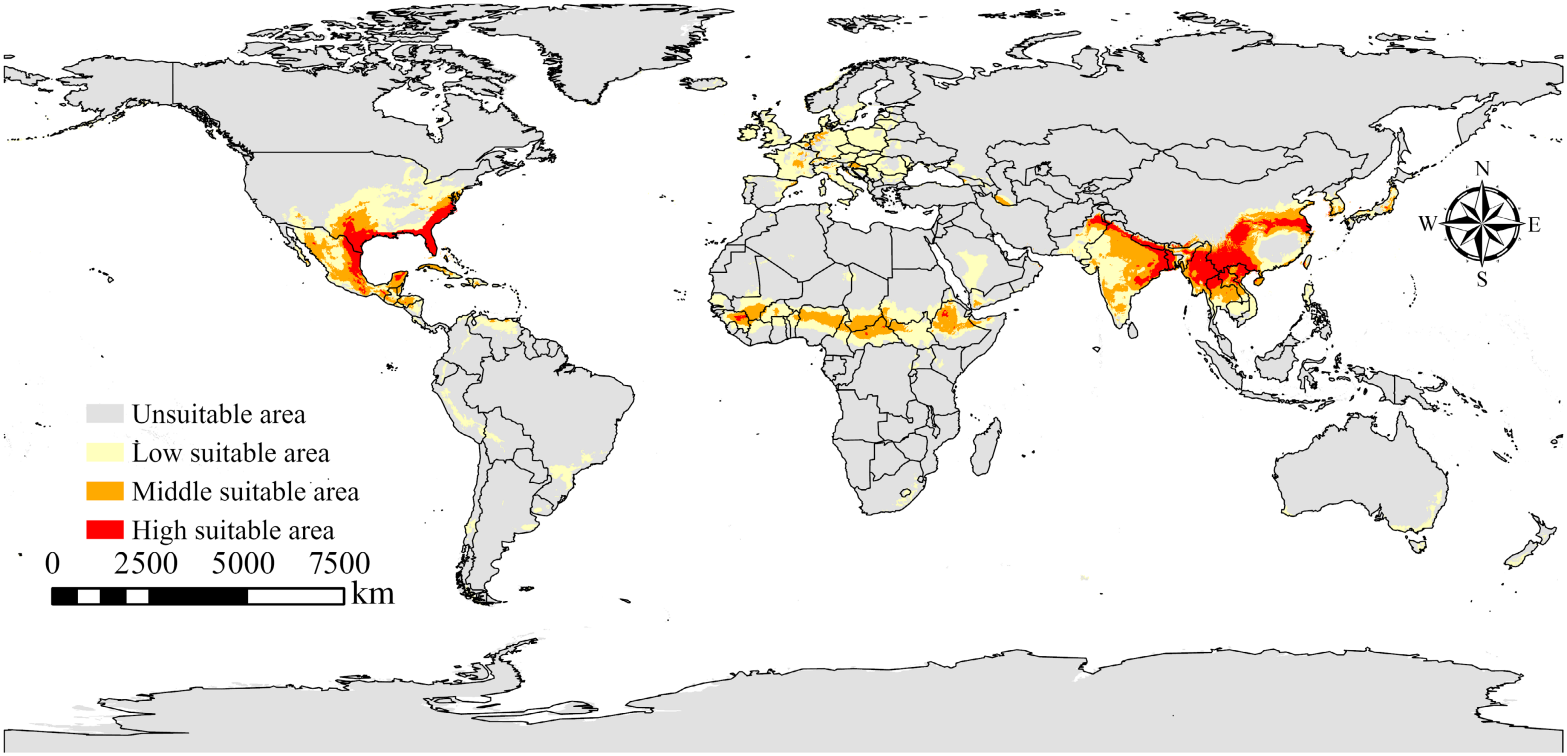
Suitable areas for *Polygenis gwyni* under current climate scenarios.

According to the results derived from MaxEnt, as illustrated in Figure 4, under current climatic conditions, the potentially suitable habitat area for species *P. simualns* spans 36.32 × 10^6^ km^2^. The areas of medium and high suitability are respectively 9.16 × 10^6^ and 4.97 × 10^6^ km^2^. These regions are predominantly located in South America, including Peru, Argentina, Chile, and Brazil; in Europe along the Mediterranean coast; in Africa, particularly in the southern parts of the continent such as South Africa and Madagascar; and in other areas of medium to high suitability encompass the Middle East, North China, and Australia. As depicted in Figure 5, under the current climate conditions, the potential suitable habitat area for species *P. gwyni* is 15.67 × 10^6^ km^2^, with the medium and high suitability areas measuring 4.01 × 10^6^ and 2.04 × 10^6^ km^2^, respectively. These regions are mainly distributed from the North China region to the Himalayan range in Asia, near the equator in Africa, and in a few parts of Europe.

### 3.3 Distribution of potentially suitable areas under future climate scenarios

The findings indicate that among 16 future climate scenarios, the total suitable habitat area for *P. simulans* increased under six scenarios, whereas for *P. gwyni*, an increase was observed under eight scenarios. See Table 3 for all areas.

**TABLE 3:** Global habitat distribution of *Pulex simulans* and *Polygenis gwyni* under current and future climate conditions (×10^6^ km^2^).

Period	Climate scenarios	Total area	Low suitability	Medium suitability	High suitability	Area change	Area change rate (%)
*P. simulans*							
1970–2000	Current	36.32	22.18	9.16	4.97	—	—
2021–2040	ssp1‐2.6	35.87	20.96	9.70	5.20	−0.45	−1.23
	ssp2‐4.5	39.07	22.55	11.07	5.45	2.75	7.58
	ssp3‐7.0	35.45	20.55	9.54	5.36	−0.87	−2.39
	ssp5‐8.5	35.47	20.71	9.47	5.29	−0.85	−2.33
2041–2060	ssp1‐2.6	35.14	20.87	9.43	4.84	−1.17	−3.23
	ssp2‐4.5	33.89	20.23	8.88	4.79	−2.42	−6.67
	ssp3‐7.0	32.77	19.04	8.89	4.84	−3.54	−9.75
	ssp5‐8.5	40.46	23.11	11.70	5.65	4.14	11.40
2061–2080	ssp1‐2.6	40.47	23.09	11.76	5.62	4.15	11.44
	ssp2‐4.5	36.92	20.97	10.42	5.54	0.61	1.67
	ssp3‐7.0	38.35	22.82	10.25	5.29	2.04	5.61
	ssp5‐8.5	35.10	20.92	9.01	5.16	−1.22	−3.36
2081–2100	ssp1‐2.6	35.60	21.04	9.43	5.13	−0.72	−1.98
	ssp2‐4.5	35.07	20.05	9.64	5.38	−1.25	−3.44
	ssp3‐7.0	37.50	22.38	9.48	5.64	1.19	3.27
	ssp5‐8.5	28.63	17.16	6.76	4.71	−7.69	−21.17
*P. gwyni*							
1970–2000	Current	15.67	9.62	4.01	2.04	—	—
2021–2040	ssp1‐2.6	17.21	10.91	4.21	2.08	1.53	9.78
	ssp2‐4.5	16.46	9.85	4.06	2.55	0.78	5.01
	ssp3‐7.0	14.59	8.51	3.68	2.40	−1.08	−6.92
	ssp5‐8.5	15.83	9.32	4.25	2.26	0.16	1.00
2041–2060	ssp1‐2.6	17.67	11.10	4.05	2.52	2.00	12.73
	ssp2‐4.5	14.05	8.43	3.81	1.81	−1.62	−10.35
	ssp3‐7.0	14.00	8.56	3.51	1.93	−1.67	−10.68
	ssp5‐8.5	19.32	12.32	4.40	2.61	3.64	23.25
2061–2080	ssp1‐2.6	14.30	8.58	3.91	1.82	−1.37	−8.74
	ssp2‐4.5	20.63	12.53	5.37	2.73	4.96	31.65
	ssp3‐7.0	15.67	10.72	3.30	1.64	−0.01	−0.04
	ssp5‐8.5	12.70	8.35	3.32	1.04	−2.97	−18.94
2081–2100	ssp1‐2.6	11.33	6.94	2.93	1.47	−4.35	−27.72
	ssp2‐4.5	13.79	8.69	3.30	1.80	−1.88	−12.01
	ssp3‐7.0	25.15	15.15	7.45	2.55	9.47	60.45
	ssp5‐8.5	18.82	12.95	4.32	1.55	3.15	20.07

**FIGURE 6:**
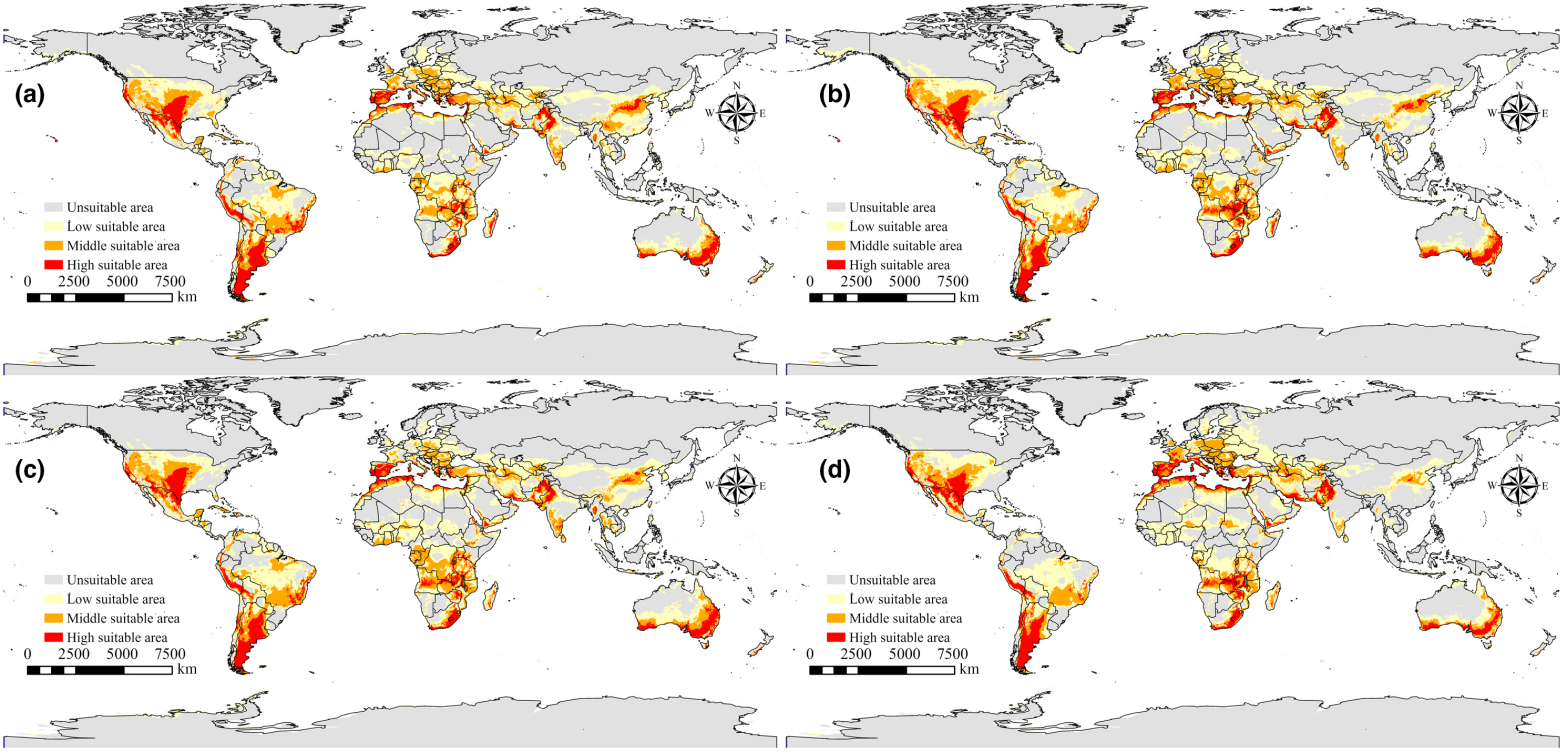
Suitable areas for *Pulex simulans* under future climate scenarios (a) SSP245:2021–2040; (b) SSP585:2041–2060; (c) SSP126:2061–2080; (d) SSP370:2081–2100.

As shown in Figure 6, the maximum area of high suitability for *P. simulans* across different time periods is observed in the SSP3‐7.0 scenario for the years 2081–2100, where the high suitability area reached its peak at 5.64 × 10^6^ km^2^, compared to the currently suitable area of 4.97 × 10^6^ km^2^. In contrast, under the SSP5‐8.5 scenario for the years 2081–2100, the high suitability area is at its minimum, measuring 4.71 × 10^6^ km^2^.

**FIGURE 7:**
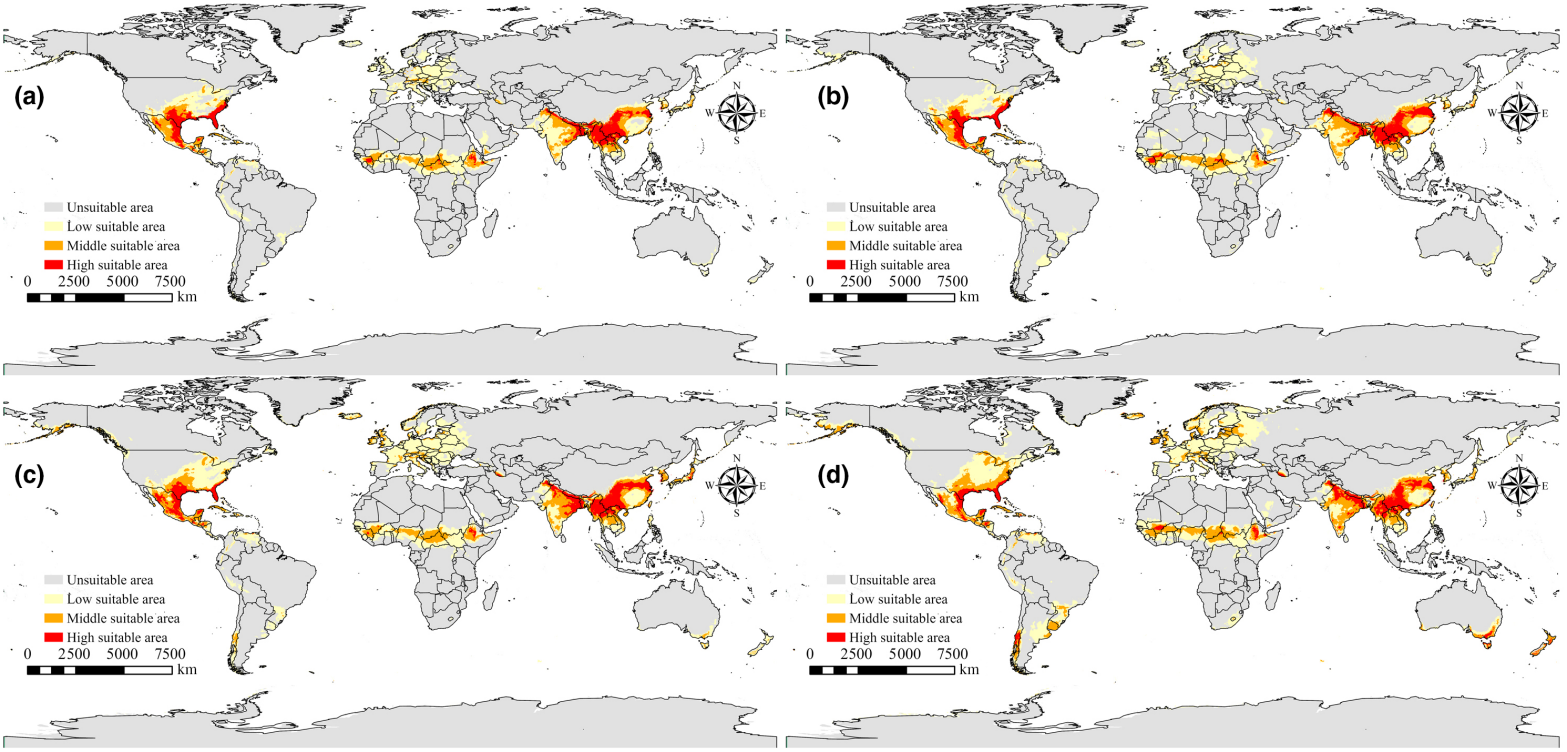
Suitable areas for *Polygenis gwyni* under future climate scenarios (a) SSP126:2021–2040; (b) SSP585:2041–2060; (c) SSP245:2061–2080; (d) SSP370:2081–2100.

For *P. gwyni*, as illustrated in Figure 7, the largest area of high suitability across different time periods is noted in the SSP2‐4.5 scenario for the years 2061–2080, where the high suitability area is the highest at 2.73 × 10^6^ km^2^, in comparison to the current suitability area of 2.04 × 10^6^ km^2^. The smallest area of high suitability is observed in the year 2061 under the SSP5‐8.5 scenario, measuring 1.04 × 10^6^ km^2^.

## 4 DISCUSSION

Fleas are vectors of some animal‐borne diseases. As the global climate changes, which significantly affects the growth and habitat range of fleas and is more likely to transmit diseases, fleas can transmit pruritic papules of the ankle, rabbit fever, cat‐scratch disease, bartonellosis, rickettsiosis, and plague (Bitam et al., 2010) and have become a problem of public health importance. We find through the model that temperature and precipitation play important roles in the distribution of these two flea species. As seen by Kreppel et al. (2016), temperature notably affects the development time of flea larvae and pupae. Fleas are highly sensitive to temperature changes, particularly during their developmental stages (van der Mescht et al., 2016), where lower temperatures in winter may reduce flea survival rates (Blagburn & Dryden, 2009; Silverman et al., 1981). Environmental stability can enhance the stability of flea populations, thereby increasing their numbers and activity. This offers more opportunities for contact with hosts, elevating the risk of biological invasions. Precipitation's impact on flea populations is equally critical; anomalous abundance of rainfall can lead to increased humidity, fostering the proliferation of fungi and mites within rodent burrows, ultimately leading to a decrease in flea populations (Krasnov, 2008; Wimsatt & Biggins, 2009).

Results obtained through Maxent clearly indicate significant changes in the distribution regions of *P. simulans* and *P. gwyni*, with such changes also dependent on various climate scenarios. Compared to *P. gwyni*, *P. simulans* has a larger area of current and future habitat; the potential habitat of *P. simulans* is South America; Europe along the Mediterranean coast; Africa, particularly in the southern parts of the continent; other areas of medium to high suitability encompass the Middle East, North China, and Australia. Differences in climate change intensity significantly influence the global distribution of their habitats, showcasing the most notable expansion during 2061–2080 under the SSP1‐2.6 scenario. The potential suitable areas for *P. gwyni* mainly encompass the North China region to the Himalayan range in Asia, regions near the equator in Africa, and several parts of Europe.

Previous studies (Li et al., 2023) have also explored the impact of climate change on the potential suitable habitats of fleas, with similarities to our study in that we both employed the Maxent model. Their research predicted the distribution of two flea species found in North America, *Peromyscopsylla hesperomys* and *Orchopeas sexdentatus*, which have distribution areas broadly similar to ours, including South America, the Mediterranean coast, and the North China region. The key difference lies in the primary environmental factors affecting the distribution of fleas; precipitation had a relatively minor impact on the flea species studied by them compared to those in our research. Earlier research by Boris R. Krasnov found that fleas in North America show the strongest response to variations in precipitation, whereas fleas in South America, Europe, and Asia respond most significantly to temperature differences (Krasnov et al., 2020).

ENMeval is the first R package to optimize the quantitative complexity assessment for MaxEnt (Kass et al., 2021). What stands out about our research lies in the use of ENMeval in R to optimize the parameters and avoid over‐fitting of the ecological niche model (ENM), resulting in more accurate results, and in the fact that the study predicts the distribution of the two fleas from a global perspective, which has broader public health implications. The weaknesses are that the ecological data do not include, e.g., normalized vegetation index, land use, relationship with hosts, etc. Young et al. (2015) find that vegetation cover negatively affects the intensity of flea infestation on hosts, which affects the intensity of flea infestation of hosts; Hieronimo et al. (2014) find that among land use types, the flea index is higher in fallow and natural forests, while the lowest flea index is found in plantation monocultures and annual mixed crops, and does not incorporate the effect of flea hosts on their abundance (Russell et al., 2018), BR Krasnov finds that the similarity between habitats of fleas composition increases with increasing similarity in hosts composition (Krasnov et al., 2006), all of which can potentially influence flea distribution and hence predictions. Despite the absence of host‐related data, we believe that the predicted distributions of *P. simulans* and *P. gwyni* are relatively consistent with their host preferences. Given the broad range of hosts for *P. simulans* (Mutlow et al., 2006), there is a potential risk of invasion; the *Rattus norvegicus*, one of the hosts for *P. gwyni*, is widely distributed globally (Worth, 1950), thus posing a potential invasion risk as well, As it is difficult to distinguish morphologically between female *P. simulans* and *P. irritans*, it may be possible to misidentify flea species to some extent, which may affect the accuracy of our predictions, we only download data in WorldClim from 1970 to 2000 under current climate conditions, which may have affected predictions due to data limitations. I believe that future research should not only consider climatic factors but also other environmental variables, and must take into account the parasitic relationships of organisms to predict the distribution of their suitable habitats.

## 5 CONCLUSIONS

We used the MaxEnt to predict the potential habitats of *P. simulans* and *P. gwyni* and found that under current climatic conditions, the medium and high suitability zones for *P. simualns* are mainly located in South America, the Mediterranean coast of Europe, the southern part of the African continent, the Middle East, North China and Australia, while the medium and high suitability zones for *P. gwyni* are mainly located in Asia, from North China to the Himalayan mountain ranges, in Africa near the equator, and in a few parts of Europe. Climate change is expanding the distribution ranges of *P. simulans* and *P. gwyni*, making it imperative to proactively address the increased risk of flea‐borne diseases driven by these changes. Enhanced surveillance in areas highly suitable for the survival of these vectors is essential to prevent disease outbreaks. Additionally, stringent border health checks should be enforced in other suitable regions to prevent the introduction of vector species. This approach is vital for mitigating the health risks associated with climate‐induced changes in the distribution of flea vectors.

## AUTHOR CONTRIBUTIONS

**Zihao Wang:** Conceptualization (equal); data curation (equal); formal analysis (equal); funding acquisition (equal); investigation (equal); methodology (equal); project administration (equal); resources (equal); software (equal); supervision (equal); validation (equal); visualization (equal); writing – original draft (equal); writing – review and editing (equal). **Nan Chang:** Funding acquisition (equal); validation (equal); writing – original draft (equal); writing – review and editing (equal). **Hongyun Li:** Conceptualization (equal); data curation (equal); software (equal). **Xiaohui Wei:** Conceptualization (equal); data curation (equal); validation (equal); writing – original draft (equal). **Yuan Shi:** Data curation (equal); methodology (equal); validation (equal). **Ke Li:** Conceptualization (equal); methodology (equal); project administration (equal). **Jinyu Li:** Data curation (equal); methodology (equal); supervision (equal). **Chenran Guo:** Investigation (equal); supervision (equal). **Qiyong Liu:** Data curation (equal); funding acquisition (equal); investigation (equal); project administration (equal); resources (equal); software (equal); supervision (equal); validation (equal); visualization (equal).

## FUNDING INFORMATION

Key R&D Program of Guangdong Province (2022B1111030002) and National Key Research and Development Program of China (2020YFC1200101).

## CONFLICT OF INTEREST STATEMENT

No competing interests declared.

## Data Availability

Climatic data used in this study can be downloaded at https://www.worldclim.org/data/cmip6/cmip6_clim5m.html. Vectors records used in this study can be downloaded at https://www.gbif.org/zh/occurrence/download?taxon_key=1419555 and https://www.gbif.org/zh/occurrence/download?taxon_key=1416419.
